# 5-ALA fluorescence in randomly selected pediatric brain tumors assessed by spectroscopy and surgical microscope

**DOI:** 10.1007/s00701-022-05360-1

**Published:** 2022-10-15

**Authors:** Peter Milos, Neda Haj-Hosseini, Jan Hillman, Karin Wårdell

**Affiliations:** 1 Department of Neurosurgery and Department of Biomedical and Clinical Sciences, Linköping University, Linköping University Hospital, 581 85 Linköping, Sweden; 2 Department of Biomedical Engineering, Linköping University, Linköping, Sweden

**Keywords:** 5-ALA, Brain tumor, Children, Spectroscopy

## Abstract

**Purpose:**

Fluorescence-guided surgery applying 5-aminolevulinic acid (5-ALA) in high-grade gliomas is an established method in adults. In children, results have so far been ambiguous. The aim of this study was to investigate 5-ALA-induced fluorescence in pediatric brain tumors by using the surgical microscope and a spectroscopic hand-held probe.

**Methods:**

Fourteen randomly selected children (age 4–17) with newly MRI-verified brain tumors were included. No selection was based on the suspected diagnosis prior to surgery. All patients received 5-ALA (20 mg /kg) either orally or via a gastric tube prior to surgery. Intratumoral fluorescence was detected with the microscope and the probe. Moreover, fluorescence in the skin of the forearm was measured. Histopathology samples revealed seven low-grade gliomas, four medulloblastomas, one diffuse intrinsic pontine glioma, one glioblastoma and one atypical meningioma. Blood samples were analyzed, and potential clinical side effects were monitored.

**Results:**

Microscopically, vague fluorescence was visible in two patients. Intratumoral fluorescence could be detected in five patients with the probe, including the two patients with vague microscopic fluorescence. Three of the oldest children had PpIX fluorescence in the skin. Nine children did not show any fluorescence in the tumor or in the skin. No clinical side effects or laboratory adverse events were observed.

**Conclusion:**

Fluorescence could not be used to guide surgery in this study, neither with the surgical microscope nor with the hand-held probe. In nine children, no fluorescence was discerned and children with noticeable fluorescence were all older than nine years. 5-ALA was considered safe to apply in children.

## Introduction

The extent of resection (EOR) in low- and high-grade gliomas is pivotal for the progression free and overall survival in both children [3, 45] and adults [21, 25, 37], recently also demonstrated irrespective of the molecular subgroup [28]. However, there is a delicate balance between achieving maximal EOR and the risk for postoperative neurological deficits, ranging between 24–44% in some studies in children, thus leading to long-term neurological and cognitive deficits [4, 13]. Great care must be taken to attain maximal resection with preservation of neurological function. Determining the tumor border zone, delineating between tumor and healthy brain tissue is thus a vital challenge for neurosurgeons. In adults, the frequency of gross total resection (GTR) is moderate, approximately 30% in high-grade gliomas (HGG) when applying standard microsurgical techniques [2]. To optimize resection, several intraoperative techniques have been developed, such as neuronavigation, intraoperative magnetic resonance imaging (MRI), intraoperative ultrasound and optical techniques using fluorescing agents [20]. Fluorescence-guided surgery implies oral or intravenous administration of a dye for visualization of fluorescence intraoperatively [46].

During the last decades, fluorescence-guided surgery using five-aminolevulinic acid (5-ALA) has been established as an intraoperative tool in HGG surgery in adults, significantly enhancing GTR [42]. Briefly, the mechanism of action is believed to be mediated through conversion of 5-ALA to protoporphyrin IX (PpIX) but not to heme, tentatively due to a downregulation of the enzyme ferrochelatase, and accumulation of PpIX in malignant tumor cells with a disrupted blood brain barrier (BBB) [12, 19, 41, 44]. Being extensively used in adults, 5-ALA is still considered an off-label product in the pediatric population. Lately, numerous studies and case reports have been published examining the role of 5-ALA in pediatric brain tumors with somewhat conflicting results, not showing a clear intraoperative benefit [1, 8–10, 14, 22–24, 32, 35, 36, 38–40, 43, 47]. Stummer et al. have previously suggested that 5-ALA should mainly be administered to children with supratentorial, strongly contrast-enhancing tumors [43]. Given the different biology of pediatric brain tumors in comparison with adults [40], these equivocal results may be conceivable. However, one also must consider the possibility of age-dependent differences in the pharmacodynamics and pharmacokinetics of 5-ALA in neonates, infants, adolescents, and adults [5, 6, 15, 26, 27].

To improve the intraoperative diagnostic accuracy of PpIX fluorescence, our group has previously developed a spectroscopic system using a hand-held probe [16], enabling detection of augmented fluorescence indicative of tumor tissue, outside the tumor margins, as identified by the fluorescence in the microscope [33]. PpIX fluorescence could also be detected in the forearm skin in adults as an indicator of potential light sensitivity [17].

The aim of this study was to examine the intraoperative PpIX fluorescence in brain tumors in children with blue-light surgical microscope and the hand-held probe system, analyzing whether it could accurately depict tumor margins. The spectroscopy system was mainly used to investigate whether the fluorescence invisible in the microscope could be detected with the probe. Another aim was to carefully monitor 5-ALA´s safety profile in children by analyzing blood tests, registering adverse events and investigating whether 5-ALA has similar light sensitivity profile in the skin as seen in adults.

## Material and methods

### Patients

Inclusion criteria were newly verified brain tumors on MR images; age 4–17 years according to decision in the ethical review board and informed, written consent by patient (when possible) and both parents. All patients referred to the center within the study time frame that fulfilled the inclusion criteria were considered for participation in the study. No selection was done based on the suspected tumor diagnosis or grade. In total 14 patients (male N = 7, female N = 7, range 4 -17 years, median age 9 years) were included in the study. Patients with recurrent tumors or planned secondary surgery were not included. All study participants had no other disease or current medications, except levetiracetam in one case of epilepsy.

Three additional patients were excluded before surgery due to blood value abnormalities. Exclusion criteria were hepatic or renal disease, known skin hypersensitivity to 5-ALA, known or first degree relative with acute or chronic porphyria, pregnancy, breast-feeding or more than 10% deviation from the normal standard lab values for liver (bilirubin, alanine aminotransferase—ALT, aspartate aminotransferase—AST, Alkaline phosphatase -ALP), kidney (Cystatin C) and hematology (C reactive protein—CRP, Reticulocytes, Leukocytes, Thrombocytes, Erythrocytes, hemoglobin – Hb, mean corpuscular hemoglobin—MCV and mean corpuscular hemoglobin concentration—MCHC) enzymes prior to surgery.

### Study protocol

**Fig. 1:**
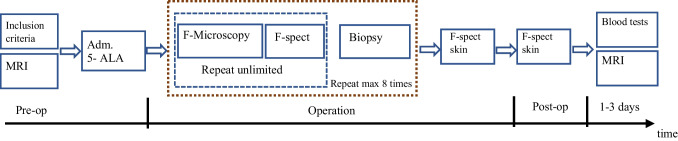
Original study design. F-spect: fluorescence spectroscopy, MRI: magnetic resonance imaging

The original study protocol is described in Fig. 1. Data was collected between September 2014 and September 2019, documented in a Case Report Form (CRF) according to Good Clinical Practice (GCP) standards. The study was monitored by a research coordinator from the regional clinical research center (Forum Östergötland). Drug approval for the clinical study was granted by the Swedish Medical Product Agency MPA (Läkemedelsverket, EudraCT: 2013–005565-40). Ethical approval was obtained from the Regional Ethical Review Board in Linköping, Sweden (Dnr 2014/350–32).

Patients were given a preoperative dose of 20 mg/kg 5-ALA, Gliolan® (Medac GmbH, Wedel, Germany) dissolved in 50–100 mL of tap water about three to four hours before induction of general anesthesia. Depending on age and cooperation, 5-ALA was administered orally or via a gastric tube in the operation room with the patient sedated. Patients were operated under general anesthesia with a combination of propofol, fentanyl and sevoflurane and placed in either prone or supine position depending on the location of the tumor. Tumor resection was performed using standardized microneurosurgical techniques together with neuronavigation (StealthStation S8, Medtronic Inc., USA) and ultrasonic aspiration (Söring GmbH, Quickborn, Germany). A surgical microscope (M720 OH5, Leica GmbH, Germany) was used during resection with the FL 400 filter option, enabling the surgeon to switch filters to detect fluorescing tissue. Spectroscopic measurements using the hand-held probe were performed in vivo under the FL 400 microscope, and ex vivo on tissue samples after resection. All operations except one were performed by the authors JH and PM. A postoperative MRI scan was performed within 48 h after surgery. Blood tests (liver, kidney and hematology) were repeated on day one and day three post-operatively (Fig. 1). Fluorescence was measured on the skin of the forearm during and after the operation.

### Fluorescence spectroscopy and the hand-held probe

A custom-made fluorescence spectroscopy system with excitation laser wavelength of 405 nm was used for measuring the fluorescence emission spectra within optical range of 450—850 nm. The laser pulse length and the spectrometer’s integration time were electronically synchronized and set to 400 ms. For skin measurements, the integration time was additionally set to 1 s to confirm spectra measured with shorter integration times. The excitation output power (10 mW) and the light collection through the fiber were calibrated before probe sterilization prior to each surgical session. The system was used together with a fiber optic hand-held probe with an outer diameter of 2 mm, a shaft length of 12 cm and a cable longer than 4 m that could extend to outside of the sterile zone in the operating room. The hand-held probe could be used as a standalone system or under the surgical microscope in the FL400 mode. The system has previously been described in detail [16, 33].

### Intraoperative fluorescence measurement procedure

Fluorescence in brain tumors was observed during surgery using both the surgical microscope and the fluorescence spectroscopy system with the fiber optic probe, separately and simultaneously with the microscope set in the FL 400 mode [33]. Three spectra were captured within 2 s for each measurement spot. One to three tissue samples from the tumor were removed and remeasured with the probe for a second time under a more controlled setup. Multiple sites on the samples were measured on and the maximum signal detected was included in the results to represent the PpIX uptake in the tumor. All observations were documented. The samples were then sent for the routine clinical histopathology examination. The overall diagnosis is included in this study, and no histopathology analysis was performed on the exact fluorescence measurement site. Since the resection was not based on fluorescence guidance, the effect of measurements on the length of the surgery was considered minimal. Measurements on the tissue samples did not affect the time of the surgery.

### Fluorescence measurements on skin

Fluorescence was measured once intra- and once postoperatively in the skin of the inner side of the forearm or on the foot or leg (when the arm could not be accessed). The postoperative measurement was performed in the postoperative ward within 24 h after 5-ALA administration. A different but similar hand-held probe was used for skin measurements with the same spectroscopy system as described above.

### Data Analysis

The fluorescence spectra were quantified by a ratiometric analysis motivated by the prior knowledge that the autofluorescence is lower in tumor and that division of PpIX fluorescence by autofluorescence would increase the contrast between tumor and non-tumor tissue in adult brain tumors [7, 18]. Moreover, this approach would reduce the variability in signals caused by the probe positioning. The *Ratio* was calculated by dividing the PpIX fluorescence intensity at wavelength of 635 nm in the spectrum by the maximum autofluorescence intensity (at approximately 510 nm). The unit of the *Ratio* is arbitrary [a.u.]. Any signal below the system’s average noise was set to zero. Data was analyzed in MATLAB® 2019–2020 (The MathWorks, Inc., Natick, MA, USA). Details of the calculation have previously been described [5]. The fluorescence intensity viewed under the surgical microscope was categorized as “none”, “vague” and “strong” based on the visual perception of the surgeon. Blood samples were analyzed with standard equipment provided by the Clinical Chemistry Department at Linköping University Hospital, Sweden.

Statistical analysis was performed using median value and non-parametric significant test (Wilcoxon signed rank test, two-tailed) in MATLAB® 2020b. The Wilcoxon signed rank test is a nonparametric test for populations with paired observations. No power calculations were performed regarding the hypothesis significance testing due to the low number of patients; therefore, the calculated p-values are only indicative of changes in the measured values.

## Results

### Patient characteristics and tumor features

Among the fourteen children undergoing primary surgery according to the study protocol, the most common initial symptoms were headache, nausea and vomiting. All patients had a preoperative Lansky score > 70. Thirteen of the tumors displayed contrast enhancement in MR images (negative, moderate or strong). Tumors were located infratentorial in nine and supratentorial in five patients. The pathology report showed medulloblastoma grade 4 (N = ), pilocytic astrocytoma grade 1 (N = 3), pilomyxoid astrocytoma grade 2 (N = 2), glioblastoma (GB) grade 4 (N = 1), oligodendroglioma grade 2 (N = 1), atypical meningioma grade 2 (N = 1), diffuse intrinsic pontine glioma (DIPG) (N = 1), desmoplastic infantile ganglioglioma grade 1 (N = 1). Ten patients underwent GTR, five patients subtotal resection (STR). Three patients developed neurological deficits post-operatively (cerebellar mutism, left leg paresis, right hemiparesis), all transient within three months. These adverse events were considered to have been caused by the surgical resection and unrelated to the administration of 5-ALA. Interestingly, these patients were all operated with STR. One patient underwent awake surgery. One patient with GB was re-operated but excluded in the study for the second operation and survived approximately two years after diagnosis. All other patients were alive in June 2020. Clinical characteristics of symptoms, tumor pathology, MRI features, location and EOR are summarized in Table 1.

**Table 1:** Clinical characteristics of patients, MRI features, tumor pathology and grade, location and EOR: Extent of Resection

PAT. NO	AGE	GENDER	WEIGHT (KG)	CONTRAST ENHANCEMENT	PATHOLOGY	LOCATION		LANSKY	EOR
#1	4Y 2 M	F	16	moderate	Medulloblastoma, gr 4	Infratentorial	vermis	70	Partial
#2	4Y 3 M	M	20	strong	Pilocytic astrocytoma gr 1	Infratentorial	vermis	70	GTR
#3	4Y 7 M	M	20	strong	Glioblastoma, gr 4	Supratentorial	temporal	80	Partial
#4	4Y 8 M	M	20	moderate	Pilomyxoid astrocytoma gr 2	Infratentorial	vermis	70	GTR
#5	4Y 10 M	M	19	strong	Pilocytic astrocytoma gr 1	Infratentorial	vermis	70	GTR
#6	5Y 10 M	M	22	strong	Desmoplastic infantile astrocytoma/ganglioglioma gr 1	Supratentorial	occipital	90	GTR
#7	5Y 11 M	F	19	strong	Medulloblastoma, gr 4	Infratentorial	vermis	80	GTR
#8	9Y 2 M	F	25	moderate	Pilocytic astrocytoma gr 1	Infratentorial	vermis	90	GTR
#9	9Y 8 M	M	31	strong	Medulloblastoma, gr 4	Infratentorial	vermis	80	GTR
#10	10Y 11 M	F	38	moderate	Pilomyxoid astrocytoma gr 2	Supratentorial	parietal	80	GTR
#11	10Y 11 M	F	36	strong	Medulloblastoma, gr 4	Infratentorial	vermis	80	GTR
#12	11Y	F	49	moderate	Diffuse intrinsic pontine glioma (DIPG)	Infratentorial	brain stem	70	Partial
#13	13Y 2 M	M	47	strong	Atypical meningioma gr 2	Supratentorial	ventricle	80	GTR
#14	17Y 1 M	M	75	negative	Oligodendroglioma gr 2	Supratentorial	frontal	90	Partial

### 5-ALA administration

5-ALA dissolved in tap water was given via a gastric tube with the patient sedated at the induction of general anesthesia to the seven youngest children (< 6 years, #1 to #7), approximately three hours before tumor resection. The 5-ALA administered via the gastric tube was flushed with 9 ml of water (the equivalent inner volume of the tube). The gastric tube was used since taste of 5-ALA is quite bitter and difficult to give to a small child. Seven children (#8 to #14) received oral 5-ALA according to standardized procedure about 3–4 h before surgery. The median surgery time was 4–5 h, well within the described peak 5-ALA fluorescence time in adults.

### Intraoperative fluorescence measurement in the microscope and with the probe

**Fig. 2:**
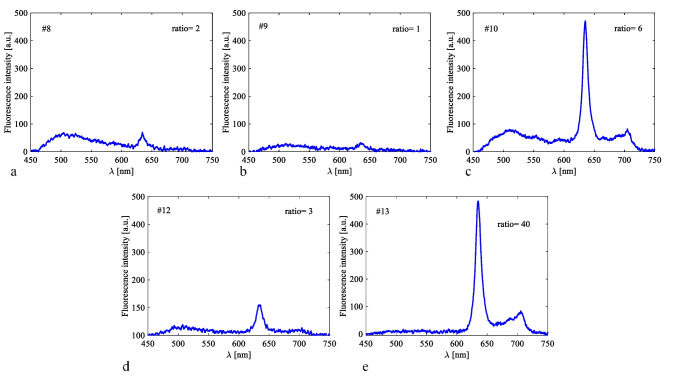
Fluorescence spectra with the highest fluorescence ratio measured in the ex vivo brain tumor tissue in five patients directly after tumor removal. The corresponding fluorescence Ratios are given in the graphs

Microscopically, there was “vague” fluorescence in two tumors (# 10 – pilomyxoid astrocytoma grade 2, #13 – atypical meningioma grade 2). The other twelve brain tumors did not reveal any visible fluorescence in the microscope. With the fiber optic hand-held probe fluorescence spectra could be detected in five tumors (# 8 – Pilocytic astrocytoma grade 1, # 9 – Medulloblastoma grade 4, # 10 – pilomyxoid astrocytoma grade 2I, #12 – diffuse intrinsic pontine glioma grade 3 (DIPG), # 13 – Atypical meningioma grade 2) (Table 2). There was no detectable fluorescence with the probe in the other nine brain tumors. Intraoperatively, neither the “vague” fluorescence in the microscope nor the spectroscopic signals provided useful guidance for the surgeon. Fluorescence spectroscopic peaks in brain tumors are shown in Fig. 2. The autofluorescence measured in pediatric brain was generally weaker than in adults making the calculated *Ratios* not directly comparable.

**Table 2:** ALA-administration and fluorescence measurement results represented by the fluorescence ratio. NA: not available. a.u.: arbitrary units

PATIENT NO	ADMIN	PROBE-TUMOR [a.u.]	MICROSCOPE- TUMOR	PROBE-SKIN INTRAOP [a.u.]	PROBE-SKIN POST OP [a.u.]
#1	tube	0	none	0	0
#2	tube	0	none	0	0
#3	tube	0	none	0	0
#4	tube	0	none	0	0
#5	tube	0	none	0	0
#6	tube	0	none	0	0
#7	tube	0	none	0	0
#8	oral	2	NA	0	0
#9	oral	1	none	0	0
#10	oral	6	vague	NA	0
#11	oral	0	none	0	0
#12	oral	3	none	0.02	0.01
#13	oral	40	vague	0.19	0.14
#14	oral	0	none	0.41	0.10

### Fluorescence measurement on the skin

**Fig. 3:**
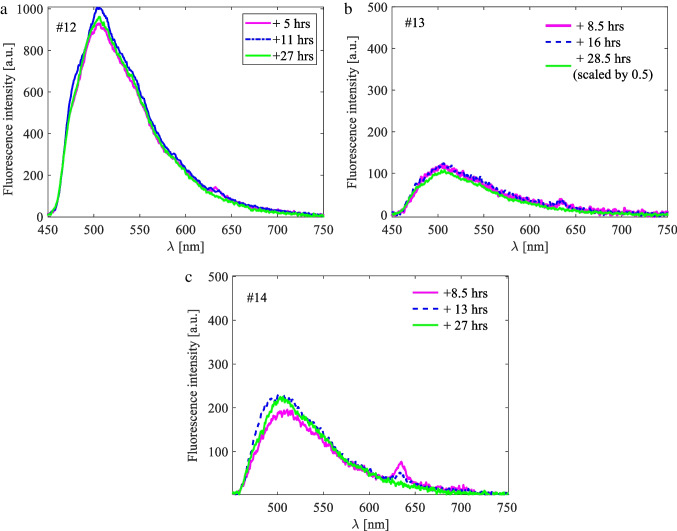
Examples of skin measurements for cases #12, #13 and #14, during operation (pink solid line), post-operatively within 24 h (dashed blue line) and 24 h after ALA administration (solid green line). The exact measurement time is stated in the graphs with reference to the time of ALA administration

Spectroscopic measurements on the skin showed PpIX fluorescence in three patients both intra- and post-operatively (# 12, #13 and #14). No fluorescence was found in ten patients during and after the operation. In one additional patient (#10) measurement was performed only after the operation since the forearm could not be accessed intraoperatively. No PpIX fluorescence could be detected in the skin of this patient. The youngest child with detectable skin fluorescence was 11 years old. The PpIX in the skin was not measurable after 24 h. Fluorescence Ratios calculated for skin measurements are small due to the low PpIX value in skin and the high skin autofluorescence (Table 2, Fig. 3).

### Blood test analysis and adverse events

**Fig. 4:**
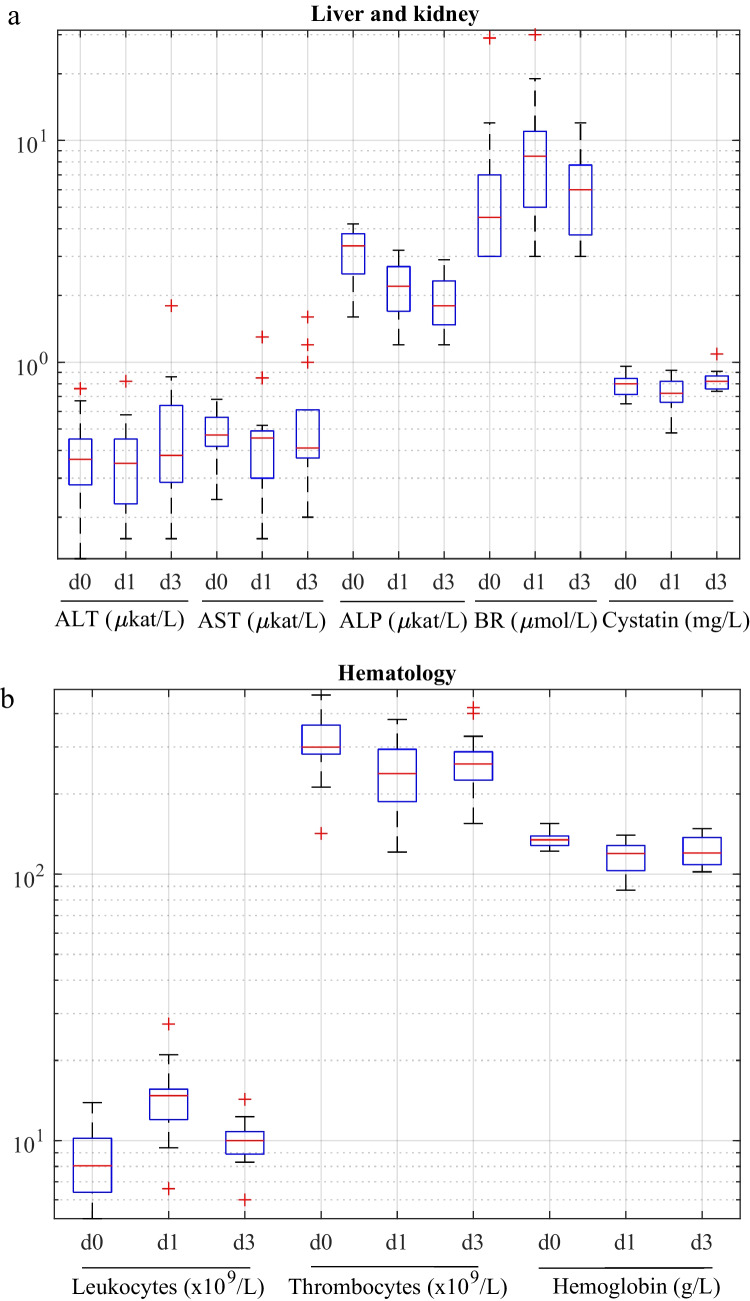
Box plots for a) the liver and kidney function, and b) hematology values. d0: prior to operation, d1: one day after the operation, d3: three days after the operation. The boxplots show the median value and the 25th-75th percentile range. ALT: alanine aminotransferase, AST: aspartate aminotransferase, ALP: Alkaline phosphatase, BR: Bilirubin, LC: Leukocytes, TC: Thrombocytes

Blood test results did not reveal any significant impact of 5-ALA on liver, kidney and hematology enzymes when comparing the whole group of children (Table 3, Fig. 4). However, values for bilirubin increased significantly (p < 0.05) on day 1, values for leukocytes increased significantly (p < 0.05) on day 1 and 3 and values for ALP, thrombocytes and Hb decreased on day 1 and 3 (p < 0.05). ALT, AST, cystatin values were statistically unchanged together with C reactive protein, reticulocytes, erythrocytes, MCV and MCHC (data not shown). All aberrant blood values in patients were eventually normalized when re-examined after approximately three weeks. No adverse or severe adverse events were observed in any patient during the study.

**Table 3:** The median and range of the blood test values prior to the operation (preop), on day 1 and day 3. P-values depict significant difference between preoperative and day 1 values, and preoperative and day 3 values. ALT: alanine aminotransferase, AST: aspartate aminotransferase, ALP: Alkaline phosphatase, BR: Bilirubin, LC: Leukocytes, TC: Thrombocytes, Hb: Hemoglobin

		ALT (µkat/L)	AST (µkat/L)	ALP (µkat/L)	BR (µmol/L)	Cystatin (mg/L)	LC (× 10^9^/ L)	TC (× 10^9^/ L)	Hb (g/L)
*Median [min, max]*	*preop*	0.37 [0.13, 0.76]	0.47 [0.24, 0.68]	3.35 [1.60, 4.20]	4.5 [3.0, 29.0]	0.8 [0.65, 0.96]	8 [5.1, 13.9]	299 [142, 470]	134 [122, 155]
	*day 1*	0.35 [0.16, 0.82]	0.46 [0.16, 1.30]	2.20 [1.20, 3.20]	8.5 [3.0, 30.0]	0.73 [0.48, 0.92]	14 [6.6, 27.4]	238 [121, 381]	119 [87, 140]
	*day 3*	0.38 [0.16, 1.8]	0.41 [0.2, 1.6]	1.8 [1.20, 2.90]	6 [3.0, 12.0]	0.82 [0.74, 1.09]	10 [6, 14.3]	259 [155, 421]	120 [102, 148]
*p-value*	*preop-day 1*			p < 0.05	p < 0.05		p < 0.05	p < 0.05	p < 0.05
	*preop-day 3*			p < 0.05			p < 0.05	p < 0.05	p < 0.05

## Discussion

Of fourteen patients, only two (# 10, # 13) displayed “vague” fluorescence in the surgical microscope intraoperatively, not sufficiently useful to guide resection. These tumors were a pilomyxoid astrocytoma grade 2 and an atypical meningioma grade 2, both located in the supratentorial region. In five patients (#8, #9, #10, #12, #13), spectroscopic fluorescence could be detected in the tumor. These included the ones with “vague” microscopic fluorescence. Two of these tumors (#9, #12) could be considered as high grade (medulloblastoma grade 4, pontine glioma grade 3), whereas the other three were low-grade tumors and meningioma (pilocytic astrocytoma grade 1, pilomyxoid astrocytoma grade 2, atypical meningioma grade 2). The three tumors with only spectroscopic fluorescence were located in the infratentorial region. Observation of fluorescence in the surgical microscope is a subjective finding depending on the experience of the surgeon. Measurements with the spectroscopic system in vivo and on multiple tissue samples provide an objective and, in our view, accurate assessment of true fluorescence [16, 18, 33, 34] as the fluorescence intensity can be quantified.

Furthermore, fluorescence in the skin was observed only in the three oldest patients in contrast to our previous findings in adults where approximately 95% had PpIX fluorescence on the forearm after receiving 5-ALA 20 mg/kg [17]. Fluorescence detection in the skin may be interpreted as an indirect sign of adequate intestinal 5-ALA uptake. However, as the autofluorescence of the skin increased with age in adults [41], it cannot be excluded that the dermal and epidermal structures could have influenced the PpIX synthesis. Nonetheless, it is remarkable that the youngest children in our study did not show any signs of fluorescence, neither in the tumor nor in the skin. Seven of the youngest children (# 1 to # 7) had been given the 5-ALA suspension through a nasogastric tube when sedated, which to our knowledge is the first time this administration of 5-ALA in children has been described in the literature. Propofol and fentanyl, also used for anesthesia in our study, have been reported to delay gastric emptying, intestinal motility and drug absorption in the small intestine [29] which may have influenced the intestinal absorption and pharmacokinetics of 5-ALA in these children. Our results are in a fair agreement with the findings in other publications where reported PpIX fluorescence in tumor seems to be more common in adolescents than in infants and toddlers [1, 8, 10, 14, 32, 35, 36, 40]. Given the lack of spectroscopic tumor and skin PpIX fluorescence in most children other tumor intrinsic or age specific factors may have contributed to the differences in PpIX fluorescence patterns in children, in comparison with adults [5, 6, 11, 15, 26, 27].

No intratumoral fluorescence was seen in the four-year-old child with a supratentorial GB. Fluorescence has previously been reported as useful in 78% of resected GBs (both primary and recurrent) in children whereas it was unhelpful or non-existent in 22% [28]. Although reported as being useful in resection of recurrent tumors [24, 39] we chose not to include recurrent GBs in our study since unspecific PpIX fluorescence may be seen in recurrent tumors originating from gliosis and reactive astrocytes and not only from tumor cells [12, 19, 30, 41, 44].

Usefulness of 5-ALA fluorescence in children has hitherto been described in a minority of cases in larger series; 47%, 44% and 43%, respectively [38, 43, 47] with predominantly high-grade histology (GB, Anaplastic Astrocytoma, Ependymoma grade II and III, Oligodendroglioma grade III, Germinoma) and supratentorial location (60% versus 40% infratentorial). However, when fluorescence was deemed helpful GTR was considerably higher than in non-fluorescing tumors (58% vs 22%) [39]. Stummer et al. have formerly advocated that 5-ALA should mainly be given to contrast enhanced, supratentorial tumors [43]. In contrast, Labuschagne recently reported two series of 23 fluorescing infratentorial (cerebellar and brain stem) tumors out of 27 [23, 24]. Among these 16 displayed “strong” fluorescence and fluorescence was considered helpful in 15 of the 27 operations (56%). The histology of tumors with “strong” fluorescence was Ependymoma grade II and III in 9 of 16 cases. Interestingly, fluorescence was not considered helpful in six tumors with “strong” fluorescence and was regarded useful in six tumors with “vague” fluorescence.

In this study thirteen tumors displayed contrast enhancement on the initial MRI scan. Only six of these where eventually diagnosed as HGG: one supratentorial (GB) and five infratentorial tumors (4 medulloblastomas, one pontine glioma grade 3), the rest were categorized as low-grade tumors and meningioma. Considering the final pathology diagnosis in all tumors, the scarcity of tumor fluorescence in our cases is conceivable. However, from a clinical viewpoint a potential HGG tumor can only be presumed preoperatively from the contrast enhancement, edema and mode of infiltration and tumor invasiveness on the MRI. The decision to use 5-ALA in adults is mainly based on these criteria [12, 19, 41, 44]. However, the larger tumor diversity in pediatric brain tumors, MRI contrast enhancement in the many LGGs but lack of contrast enhancement in some HGGs [31] makes it more difficult to adapt a clear preoperative algorithm for the administration of 5-ALA in pediatric patients. From the initial, diagnostic MRI scans we had expected more cases of HGGs and detection of tumor fluorescence, especially with the spectroscopic probe system.

No clinical side effects or adverse effects from 5-ALA were noted during the study. Transient elevations of blood samples were observed in some patients but eventually normalized and did not warrant any medical measures.

This study comprises a small number of patients with different tumor histopathology, mainly low-grade tumors, making it difficult to properly discern the potential usefulness of 5-ALA in a series consisting of more HGGs. However, thirteen out of fourteen tumors were contrast enhancing on preoperative MRI, mimicking a possible high-grade glioma, and thus justifying inclusion in the study.

## Conclusions

Five of fourteen tumors showed PpIX fluorescence. Microscopic fluorescence was “vague” in two patients and not useful to guide tumor resection in our study. The hand-held probe revealed fluorescence in additional three tumors and in the skin in the three oldest children, resembling our previous results in adults. Children displaying fluorescence were all older than nine years. 5-ALA appear to be safe for use in children older than four years and in adolescents.
